# International Migration of Doctors, and Its Impact on Availability of Psychiatrists in Low and Middle Income Countries

**DOI:** 10.1371/journal.pone.0009049

**Published:** 2010-02-04

**Authors:** Rachel Jenkins, Robert Kydd, Paul Mullen, Kenneth Thomson, James Sculley, Susan Kuper, Joanna Carroll, Oye Gureje, Simon Hatcher, Sharon Brownie, Christopher Carroll, Sheila Hollins, Mai Luen Wong

**Affiliations:** 1 Health Services and Population Research Department, Institute of Psychiatry, King's College London, London, United Kingdom; 2 Department of Psychiatry and Behavioral Science, University of Auckland, Auckland, New Zealand; 3 Department of Forensic Science, Monash University, Victoria, Australia; 4 Department of Psychiatry, University of Pittsburgh, Pittsburgh, Pennsylvania, United States of America; 5 Department of Neuropsychiatry and Behavioral Science, South Carolina School of Medicine, Columbia, South Carolina, United States of America; 6 Department of Memberships, American Psychiatric Association, Arlington, Virginia, United States of America; 7 Department of International Affairs, Royal College of Psychiatrists, London, United Kingdom; 8 Department of Psychiatry, University of Ibadan, Ibadan, Nigeria; 9 Department of Medical and Health Sciences, University of Auckland, Auckland, New Zealand; 10 Royal Australian and New Zealand College of Psychiatrists, Canberra, Australia; 11 US Department of Health and Human Science, Washington, D.C., United States of America; 12 Deanery Department, Royal College of Psychiatrists, London, United Kingdom; University of Western Sydney, Australia

## Abstract

**Background:**

Migration of health professionals from low and middle income countries to rich countries is a large scale and long-standing phenomenon, which is detrimental to the health systems in the donor countries. We sought to explore the extent of psychiatric migration.

**Methods:**

In our study, we use the respective professional databases in each country to establish the numbers of psychiatrists currently registered in the UK, US, New Zealand, and Australia who originate from other countries. We also estimate the impact of this migration on the psychiatrist population ratios in the donor countries.

**Findings:**

We document large numbers of psychiatrists currently registered in the UK, US, New Zealand and Australia originating from India (4687 psychiatrists), Pakistan (1158), Bangladesh (149) , Nigeria (384) , Egypt (484), Sri Lanka (142), Philippines (1593). For some countries of origin, the numbers of psychiatrists currently registered within high-income countries' professional databases are very small (e.g., 5 psychiatrists of Tanzanian origin registered in the 4 high-income countries we studied), but this number is very significant compared to the 15 psychiatrists currently registered in Tanzania). Without such emigration, many countries would have more than double the number of psychiatrists per 100, 000 population (e.g. Bangladesh, Myanmar, Afghanistan, Egypt, Syria, Lebanon); and some countries would have had five to eight times more psychiatrists per 100,000 (e.g. Philippines, Pakistan, Sri Lanka, Liberia, Nigeria and Zambia).

**Conclusions:**

Large numbers of psychiatrists originating from key low and middle income countries are currently registered in the UK, US, New Zealand and Australia, with concomitant impact on the psychiatrist/population ratio n the originating countries. We suggest that creative international policy approaches are needed to ensure the individual migration rights of health professionals do not compromise societal population rights to health, and that there are public and fair agreements between countries within an internationally agreed framework.

## Introduction

Migration of professionals from low and middle income countries to richer countries is a large scale phenomenon, and in 2000 it was estimated that there were 1.5 million professionals from developing countries working in industrialised countries[1]. Certain sets of skills and competencies are so specialised and in such short supply that they are being sourced on a global basis[2]. This global movement includes doctors and nurses, and the loss of such health resources for developing countries results in a loss of capacity of the health system to deliver health care equitably [3]. Thus health worker migration matters because human resources are fundamental to the delivery of health services, and explicit human resource policies are crucial for the implementation of health sector reforms in low and middle income countries, and for the achievement of the Millennium Development Goals[4]. Although human resources for health in low and middle income countries should have been greatly expanded in the last 50 years, in practice, despite the establishment of and considerable efforts by universities and training colleges, there have been substantial losses through brain drain to the richer countries, who derive significant proportions of their health workforce from poorer countries [5], [6], [7], [8]. Governments in low and middle income countries and other observers have expressed considerable concern about the impact of such migration on their health systems [6], [9], [10], [11] , and clear arguments for strengthening human resources for health in poor countries have been made [12].

The factors influencing migration, namely the so-called push and pull factors, have been explored[13], [14], [15], [16] .

Push factors include low salaries, poor occupational safety (especially in relation to HIV infection), inadequacy of facilities and supply of medicines, lack of post graduate training and continuing professional development and an expectation and practice within some universities in low income countries of encouraging graduates to go abroad. Pull factors include both active and passive recruitment by high income countries, job vacancies in high income countries with concomitant high salaries, better working conditions and facilities, and better access to higher training and continuing professional development. In specialist areas where there is significant brain drain, overload and professional isolation in those health workers left behind may encourage further emigration.

Taking the UK as an example of a recipient country, the UK has a long tradition of recruiting doctors and nurses from the Commonwealth [17], [18]. Thus there was a rapid growth in inflow of nurses to the UK, rising from 3,621 in 1998 to 16,000 entering in 2002/2. In 2004/5, over half of the 12000 nurses entering the UK were from India and the Philippines. The NHS stopped active recruitment in 2005/6, [18]. However, the private sector is still actively recruiting, and there is a flow of overseas trained nurses from the private sector to the NHS. Thus in 2007, 6314 overseas trained nurses gained initial registration [12]. Nursing influx to the US has also grown in recent years, rising to approximately 15,000 per annum, half from the Philippines, and significant proportions from India, Nigeria, Jamaican, South Africa and Ghana. Ireland is also a major destination country for nurses [18], [19], [20]. Nurse migration has had significant impact on the experience and seniority of staff remaining in hospitals in the Philippines, most of whom are now under 40 [21].

Medical flows to the UK have been less well documented than nursing flows. The General Medical Council (GMC) reported a rise of 38% in overseas trained doctors registering in UK between 1993 and 2002. In 2008, out of a total of 245,067 doctors registered with the GMC, 91,982 of these are foreign medical graduates. In the US, 23% of doctors trained overseas and 64% of these came from low and middle income countries [22]. Thus, 5334 doctors from Sub-Saharan Africa are now in the US, of which 86% are from Nigeria, South Africa and Ghana [23]. Altogether there are 10,936 doctors in the US, UK and Canada from Sub-Saharan Africa, which is 12% of all currently employed/working African educated physicians.

There are marked differences between medical specialties both in terms of the absolute numbers of professionals moving from poorer to richer countries, and also in the impact of the loss of services to the poorer countries, and it is therefore of interest to examine the situation in relation to psychiatry.

Mental health services are particularly scarce in low and middle income countries, and scaling up mental health services is especially challenging, requiring a strategic approach to human resource development, retention and deployment in low and middle income countries [24]. For example, the recent Lancet series on mental health identified low numbers of mental health specialists as a barrier to the improvement of mental health services in low-income and middle-income countries with the deficit reflecting not only on the delivery of specialist services but also on needed training and supervision of primary and general health care workers[25]. A number of authors have already expressed concern about brain drain specifically in relation to psychiatrists [26], [27]. It is therefore crucial to collect empirical data to understand the scale of this brain drain as psychiatrists and psychiatric nurses are essential not only for the delivery of specialist services, but even more importantly for the support and supervision of front line primary care workers, for intersectoral liaison with the education, social welfare and criminal justice systems, and for training, service development and leadership at district, provincial and national levels[28], [29]. Obtaining empirical data on the movement of mental health workers to developed countries will assist the recent proposal [25], addressed to those developed countries receiving migrant health workers, to provide significant resources to assist mental health services in those countries from which the workers have come [30].

This paper therefore aims to assess the scale of the brain drain of psychiatrists to four high-income countries - UK, US, Australia and New Zealand - and to estimate its impact on the population ratios of psychiatrists in donor countries. The first three of these countries were specifically identified in the recent call for action published in *Lancet* [25], and these countries were suggested to be the main beneficiaries of the brain drain of mental health professionals from low-income and middle-income countries. It is therefore important to document the extent of the mental health manpower resources from which they have benefitted in order to determine what level of response is required from them. This response could subsequently form part of the planned monitoring of the implementation of the call[30].

## Methods

Data on migrant psychiatrists with specialist qualifications were obtained from the professional association of psychiatrists in each of the participating countries, Australia, New Zealand, UK and USA.

In the UK, the Royal College of Psychiatrists 2007 databases for England and Wales, Scotland and Ireland were analysed to provide data on members whose specialist qualifications have been obtained in other countries.

In New Zealand, the data was obtained from the 2006 Medical Council of New Zealand annual workforce survey. This is a compulsory survey of all doctors working in New Zealand who complete it as part of their annual registration application. It provides data on the country of origin of the basic medical degree for specialists (Consultants), registrars (Interns) and medical officers. The figures we report from New Zealand are for Consultants only.

In Australia, registry data from 2008 was obtained from the Royal Australian and New Zealand College of Psychiatrists (RANZCP). It takes into account Fellows of the College and Exemptions candidates applying for fellowship who obtained their specialist qualifications outside of Australia.

We were not able to obtain data on the group of overseas trained specialist psychiatrists in Australia who are practicing in public health services in an AON (Area of Need), i.e., in rural and outer metropolitan areas but who are not Fellows of the RANZCP.

Investigation revealed these data are not held by the RANZCP or any state bodies, but rather by the Australian Institute of Health and Welfare, which planned to release the 2006 AIHW Medical Labour Force report in late 2008, making it unavailable at the time of analysis.

In the USA, the 2008 data were obtained from the American Psychiatric Association, of which most psychiatrists are members. It provides data on the country of origin of their basic medical degree.

Data were grouped into countries of origin, classified by WHO regions to calculate the numbers who have moved from each source country to each of the four western countries under consideration, namely UK, US, Australia and New Zealand. The WHO Mental Health Atlas 2005, a compendium of government reported data on mental health, was then used to gather data on the population of each country of origin (A) and the number of psychiatrists per 100,000 (B). These figures were used to calculate the number of psychiatrists in each of the countries of original qualification, collected as above [(AxB)/100, 000 = C].

The impact of the brain drain on that country (“impact factor”) was then calculated by adding the number of psychiatrists (C) each participating country had and re-calculating the number of psychiatrists per 100,000 there might have been without emigration [(100000xC)/A].

## Results

The tables show the number of psychiatrists for each source country in each of the six WHO regions who are now working in the UK, Australia, New Zealand and the US, the population of the source country, the number of psychiatrists in that country, the ratio of psychiatrists per 100,000, and the impact of emigration. Table 1 demonstrates the US draws very significant numbers of doctors who are either psychiatrists or who later become psychiatrists from the low and middle income countries in the WHO Americas region, the UK draws a few psychiatrists, and Australia and NZ only draw from the high income countries. The majority of migrant psychiatrists working in the UK who originate from the WHO Americas are drawn from a few specific countries in the region, namely Argentina, Brazil, Canada, Cuba, Dominican Republic, Jamaica, Trinidad, USA and Venezuela, while in NZ they come entirely from the US, and in Australia they come in roughly equal numbers from Canada and the US. The US lists nearly 3000 psychiatrists who originally come from the Americas region, somewhat surprisingly none from Canada, but nearly 800 are from Mexico, nearly 600 from the Dominican Republic, 250 are from Cuba and over 200 each are from Columbia and Grenada, and nearly 100 each from Brazil and Peru, and smaller numbers from a wide range of other countries. However, it should be noted that a significant proportion of the doctors apparently originating from Mexico, Grenada and the Dominican Republic would in fact be US citizens studying in medical schools in those countries and then returning to work in the US [31]. Further, excess Cuban doctors are trained for deployment abroad as a foreign policy strategy.

**Table 1:** The number of psychiatrists with specialist qualifications from each of the countries in the WHO Americas Region who are now working in the UK, Australia, New Zealand and USA.

Country of origin	No. in UK^1^	No. in Australia^2^	No. in NZ^3^	No. in USA^4^	Population of country^5^	Psychiatrists per 100,000 population^5^	Psychiatrists per 100,000 population if there had been no brain drain	Psychiatrists remaining in that country	Psychiatrists remaining if there had been no brain drain
Antigua				6	77000	2.00	9.79	2	8
Argentina	1	1		18	38871000	13.25	13.30	5150	5170
Belize				3	261000	1.30	2.45	3	6
Bolivia				8	8973000	0.90	0.99	81	89
Brazil	8	1		96	180655000	4.80	4.86	8671	8776
Canada	7	29			31743000	12.00	12.11	3809	3845
Chile				47	15997000	4.00	4.29	640	687
Columbia				228	44914000	2.00	2.51	898	1126
Costa Rica				21	4250000	2.00	2.49	85	106
Cuba	2			250	11328000	10.00	12.22	1133	1385
Dominican Republic	1			589	8873000	2.00	8.65	177	767
Ecuador				23	13193000	2.10	2.27	277	300
El Salvador				21	6614000	0.50	0.82	33	54
Grenada				208	103000	1.00	202.94	1	209
Guatemala				51	12661000	0.54	0.94	68	119
Honduras				8	7100000	0.76	0.87	54	62
Jamaica	2			29	2676000	1.60	2.76	43	74
Mexico				792	104931000	2.70	3.45	2833	3625
Nicaragua				15	5596000	0.64	0.91	36	51
Panama				9	3178000	3.70	3.98	118	127
Paraguay				11	6018000	1.80	1.98	108	119
Peru				90	27567000	2.06	2.39	568	658

Table 2 shows that both the US and UK databases list high numbers of psychiatrists originating from the Southeast Asia region (1302 to the UK and 3680 to the US, both largely drawn from India). Other countries besides India that have contributed psychiatrists to the UK are Bangladesh, Myanmar, Nepal and Sri Lanka. The US has received 3293 from India, 106 from Sri Lanka and 78 from Myanmar. New Zealand has received 13 from India, and Australia has received 150 from the region, of whom 137 are from India, and 13 from Sri Lanka. Table 2 also shows that the ratio of psychiatrists per 100,000 population is estimated to be almost five times higher in Sri Lanka, three times higher in Bangladesh, and twice as high in Myanmar if those currently working abroad had instead continued their psychiatric careers in their original countries.

**Table 2:** The number of psychiatrists with specialist qualifications from each of the countries in the WHO South-East Asia Region who are now working in the UK, Australia, New Zealand and USA.

Country of origin	No. in UK^1^	No. in Australia^2^	No. in NZ^3^	No. in USA^4^	Population of country^5^	Psychiatrists per 100,000 population^5^	Psychiatrists per 100,000 population if there had been no brain drain	Psychiatrists remaining in that country	Psychiatrists remaining if there had been no brain drain
Bangladesh	15			134	149665000	0.05	0.15	75	224
India	1235	137	22	3293	1081000000	0.20	0.63	2162	6849
Indonesia				28	222611000	0.21	0.22	467	495
Myanmar	25			78	50101000	0.20	0.41	100	203
Nepal	4			7	25724000	0.12	0.16	31	42
Sri Lankar	23	13		106	19218000	0.20	0.94	38	180
Thailand				34	63465000	0.60	0.65	381	415
Total	1302	150	22	3680					

1 Data from The Royal College of Psychiatrists 2007

2 Data from RANZCP 2008

3 Data from 2006 Medical Council of New Zealand annual workforce survey

4 Data from the American Psychiatric Association 2008

5 Data from the WHO Atlas 2005

Table 3 shows the UK lists 467 psychiatrists originally from the Africa region (214 from Nigeria, 196 from South Africa, a few from Ghana and a handful from several other countries), while the US has received 383 psychiatrists from Africa, again mostly from Nigeria and South Africa. In NZ, all come from South Africa, and in Australia, nearly all come from South Africa with one exception from Uganda. We estimate the ratio of psychiatrists per 100,000 population would be between 2 and 8 times higher in The Congo, Ghana, Liberia, Nigeria, South Africa, Zambia, Zimbabwe those currently working abroad had instead continued their psychiatric careers in their original countries. The Uganda data in WHO Atlas is anomalous as in fact Uganda only has 23 psychiatrists ; it is probable that Uganda, in reporting to WHO Atlas, included the important cadre of medical officers trained in psychiatry (who have had a three year basic medical training rather than the normal five or six years).

**Table 3:** The number of psychiatrists with specialist qualifications from each of the countries in the WHO African Region who are now working in the UK, Australia, New Zealand and USA.

Country of origin	No. in UK^1^	No. in Australia^2^	No. in NZ^3^	No. in USA^4^	Population of country^5^	Psychiatrists per 100,000 population^5^	Psychiatrists per 100,000 population if there had been no brain drain	Psychiatrists remaining in that country	Psychiatrists remaining if there had been no brain drain
Algeria				8	32339000	1.10	1.12	356	364
Cameroon				2	16296000	0.03	0.04	5	7
Congo				1	3818000	0.03	0.06	1	2
Ethiopia	3			6	72420000	0.02	0.03	14	23
Ghana	16			18	21377000	0.08	0.24	17	51
Guinea				1	8620000	0.04	0.05	3	4
Kenya	9			3	32420000	0.20	0.24	65	77
Liberia				5	3487000	0.03	0.17	1	6
Malawi	1				12337000	0.00	0.01	0	1
Mauritius	1				1233000	1.00	1.08	12	13
Nigeria	214	3		167	127117000	0.09	0.39	114	498
Senegal				4	10339000	0.16	0.20	17	21
Sierra Leone				6	5169000	0.02	0.14	1	7
South Africa	196	23	38	144	45214000	1.20	2.09	543	944
Tanzania	4			1	37671000	0.04	0.05	15	20
Uganda	4	1		8	26699000	1.60	1.65	23	46
Zambia	9			6	10924000	0.02	0.16	2	17
Zimbabwe	10			3	12932000	0.10	0.20	13	26
Total	467	27	38	383					

1 Data from The Royal College of Psychiatrists 2007

2 Data from RANZCP 2008

3 Data from 2006 Medical Council of New Zealand annual workforce survey

4 Data from the American Psychiatric Association 2008

5 Data from the WHO Atlas 2005

Table 4 shows the 1845 US psychiatrists originate from the WHO Eastern Mediterranean region, of whom 972 are from Pakistan, 382 from Egypt, 91 from Lebanon, 73 from Syria, 46 from Iraq and 18 from Afghanistan.

**Table 4:** The number of psychiatrists with specialist qualifications from each of the countries in the WHO Eastern Mediterranean Region who are now working in the UK, Australia, New Zealand and USA.

Country of origin	No. in UK^1^	No. in Australia^2^	No. in NZ^3^	No. in USA^4^	Population of country^5^	Psychiatrists per 100,000 population^5^	Psychiatrists per 100,000 population if there had been no brain drain	Psychiatrists remaining in that country	Psychiatrists remaining if there had been no brain drain
Afghanistan	1			18	24926000	0.04	0.11	9	28
Bahrain	1				739000	5.00	5.14	37	38
Egypt	100	2		382	73389000	0.90	1.56	661	1145
Iran	27	3		238	69789000	1.90	2.28	1326	1594
Iraq	67	3		46	25856000	0.70	1.15	181	297
Jordan	1			8	5613000	1.00	1.16	56	65
Kuwait	1			2	2595000	3.10	3.22	80	83
Lebanon	3			91	3708000	2.00	4.54	74	168
Libya	2				5659000	0.18	0.22	10	12
Morocco				7	31064000	0.40	0.42	124	131
Oman	1				2935000	1.40	1.43	41	42
Pakistan	181	1	4	972	157315000	0.20	0.94	315	1473
Saudi Arabia	4			5	24919000	1.10	1.14	274	283
Sudan	28			3	34333000	0.09	0.18	31	62
Syria	4			73	18223000	0.50	0.92	91	168
UAE	2				3051000	2.00	2.07	61	63
Total	423	9	4	1845					

1 Data from The Royal College of Psychiatrists 2007

2 Data from RANZCP 2008

3 Data from 2006 Medical Council of New Zealand annual workforce survey

4 Data from the American Psychiatric Association 2008

5 Data from the WHO Atlas 2005

423 psychiatrists registered in the UK originate from WHO Eastern Mediterranean region, of whom 181 are from Pakistan, 67 from Iraq, 27 from Iran and 28 from Sudan. NZ draws very few from this region (only 4 from Pakistan), as does Australia with only 3 from Iran and 3 from Iraq. We estimate the ratio of psychiatrists per 100,000 population in Pakistan would be 4 to 5 times higher had the psychiatrists from Pakistan currently registered abroad continued to work in psychiatry in their country of origin, and would be 1.5–3 times higher in Afghanistan, Egypt, Lebanon and Syria.

Table 5 shows the US has received numbers (2322) from the WHO Pacific region, of whom 1590 are from the Philippines, 476 from Korea, 86 from Cambodia, 77 from Australia, 44 from Japan, and 34 from Vietnam. The UK only draws 64 from this region, of whom 29 are from Australia, 17 from NZ, 7 from Singapore and 5 from China. New Zealand currently does not have any psychiatrists who originate from this region, while the Australian register lists a large number originating from New Zealand (151), with 4 from Malaysia and 1 from Singapore. The table indicates that the greatest relative depletion of psychiatrist population ratio is in the Philippines where it would be six times higher if those currently working abroad had instead continued their psychiatric careers in the Philippines, and interestingly in New Zealand which would have nearly twice the number of psychiatrists if those currently working abroad had instead continued their psychiatric careers in New Zealand.

**Table 5:** The number of psychiatrists with specialist qualifications from each of the countries in the WHO Western Pacific Region who are now working in the UK, Australia, New Zealand and USA.

Country of origin	No. in UK^1^	No. in Australia^2^	No. in NZ^3^	No. in USA^4^	Population of country^5^	Psychiatrists per 100,000 population^5^	Psychiatrists per 100,000 population if there had been no brain drain	Psychiatrists remaining in that country	Psychiatrists remaining if there had been no brain drain
Australia	29			77	19913000	14.00	14.53	2788	2894
Cambodia				1	14482000	0.16	0.17	23	24
China	5			86	1313000000	1.29	1.30	16938	17029
Fiji				1	847000	0.25	0.37	2	3
Japan				44	127799000	9.40	9.43	12013	12057
Malaysia	3	4		2	24876000	0.60	0.64	149	158
New Zealand	17	151		11	3905000	6.60	11.18	258	437
Philippines	3			1590	81408000	0.40	2.36	326	1919
Republic of Korea				476	47950000	3.50	4.49	1678	2154
Singapore	7	1			4315000	2.30	2.49	99	107
Vietnam				34	82481000	0.32	0.36	264	298
Total	64	156		2322					

1 Data from The Royal College of Psychiatrists 2007

2 Data from RANZCP 2008

3 Data from 2006 Medical Council of New Zealand annual workforce survey

4 Data from the American Psychiatric Association 2008

5 Data from the WHO Atlas 2005

Table 6 shows that the UK currently employs psychiatrists originating from a wide range of European countries, with the majority from Ireland, Spain, Germany, Russia, and Poland.

**Table 6:** The number of psychiatrists with specialist qualifications from each of the countries in the WHO European Region who are now working in the UK, Australia, New Zealand and USA.

Country of origin	No. in UK^1^	No. in Australia^2^	No. in NZ^3^	No. in USA^4^	Population of country^5^	Psychiatrists per 100,000 population^5^	Psychiatrists per 100,000 population if there had been no brain drain	Psychiatrists remaining in that country	Psychiatrists remaining if there had been no brain drain
Albania				3	3193000	2.20	2.29	70	73
Armenia	2			27	3052000	4.00	4.95	122	151
Austria	6	1		55	8120000	11.80	12.56	958	1020
Azerbaijan				21	8447000	5.00	5.25	422	443
Belarus				14	9851000	10.10	10.24	995	1009
Belgium	11			116	10339000	18.00	19.23	1861	1988
Bosnia & Herzegovina	2			27	4186000	1.80	2.49	75	104
Bulgaria	11	4		35	7829000	9.00	9.64	705	755
Croatia	3			63	4416000	8.70	10.19	384	450
Cyprus	2				807000	5.00	5.25	40	42
Czech Republic	7			43	10226000	12.10	12.59	1237	1287
Denmark	3	1		2	5375000	16.00	16.09	860	866
Estonia				1	1308000	13.00	13.08	170	171
Finland	1	1		8	5216000	22.00	22.19	1148	1158
France	7	2		92	60434000	22.00	22.17	13295	13396
Georgia				14	5074000	6.00	6.28	304	318
Germany	76	4		247	82526000	11.80	12.20	9738	10065
Greece	31			83	10977000	15.00	16.04	1647	1761
Hungary	6	6		98	9831000	9.00	10.12	885	995
Iceland				4	291000	25.00	26.37	73	77
Ireland	150	11		139	3999000	6.82	14.32	273	573
Israel		1		188	6560000	13.70	16.58	899	1088
Italy		2		296	57346000	9.80	10.32	5620	5918
Kazakhstan				7	15403000	6.00	6.05	924	931
Kyrgyzstan				2	5208000	4.50	4.54	234	236
Latvia				34	2286000	10.00	11.49	229	263
Lithuania				12	3422000	15.00	15.35	513	525
Macedonia	7			7	2066000	7.50	8.18	155	169
Malta	16			2	396000	4.00	8.55	16	34
Moldova				13	4263000	9.00	9.30	384	397
Netherlands	12	4		71	16227000	9.00	9.54	1460	1547
Norway				8	4552000	20.00	20.18	910	918
Poland	24			219	38551000	6.00	6.63	2313	2556
Portugal	1			11	10072000	4.70	4.82	473	485
Romania	17	1		264	22280000	4.10	5.37	913	1195
Russian Federation	25			350	142397000	13.30	13.56	18939	19314
Serbia	1			67	10519000	12.80	13.45	1346	1414
Slovakia	2			12	5407000	10.00	10.26	541	555
Slovenia				5	1982000	5.35	5.60	106	111
Spain	88			352	41128000	3.60	4.67	1481	1921
Sweden	2			17	8886000	20.00	20.21	1777	1796
Switzerland	2	1		139	7163000	23.00	24.98	1647	1789
Tajikistan				7	6297000	1.80	1.91	113	120
Turkey	6	3		199	72320000	1.00	1.29	723	931
Turkmenistan				1	4940000	3.00	3.02	148	149
Ukraine	14	1		130	4815100	8.90	11.91	429	574
United Kingdom		333	63	216	59428000	11.00	12.03	6537	7149
Uzbekistan				42	26479000	3.30	3.46	874	916
Total	535	376	63	3763					

1 Data from The Royal College of Psychiatrists 2007

2 Data from RANZCP 2008

3 Data from 2006 Medical Council of New Zealand annual workforce survey

4 Data from the American Psychiatric Association 2008

5 Data from the WHO Atlas 2005

New Zealand has drawn 63, all from the UK. Australia has taken 333 from the UK, and small numbers from each of 14 other European countries. The US has attracted 3763 psychiatrists from across Europe, of whom only 216 are from the UK, and the rest are from a very wide spread of countries. Ireland, Israel and Malta are the countries that have suffered most from brain drain in the European region, halving the psychiatrist population ratios for Ireland and Malta.

### Conclusion

We document large numbers of psychiatrists currently registered in high income countries who originate from low and middle income countries. Some countries have experienced a high volume of outward migration in terms of sheer numbers (e.g. India, Pakistan, Bangladesh, Nigeria, Egypt, Sri Lanka, Philippines), while other countries have only lost a few psychiatrists, but these few represent a large proportion of the country total, (e.g. Tanzania) . Thus we estimate that many countries would have probably have more than double the proportion of psychiatrists per 100,000 (e.g. Bangladesh, Myanmar, Afghanistan, Egypt Syria, Lebanon), and some countries would have five-eight times more psychiatrists per 100,000 if those currently working abroad had instead continued their psychiatric careers in their original countries (e.g. Philippines, Pakistan, Sri Lanka, Liberia, Nigeria, Zambia).

There are several limitations to our data. Firstly, the professional psychiatric associations in each participating country collect data in a different way, with the UK and Australia collecting data on the members' psychiatric as well as basic medical qualifications from their country of origin, and the US and NZ collecting data on basic medical qualification from the country of origin.

Secondly, not all specialist-trained psychiatrists who migrate are registered and practicing in the field in which they are trained, and are thus not accounted for by the data provided by the professional associations.

Thirdly, data provided by the professional associations for this analysis are for people who are currently registered with their respective professional associations, using information supplied when the individual registered, whereas data from the WHO Atlas is from 2005. Although the basic and psychiatric qualifications would not be expected to change over time, nonetheless it is unknown how much of a difference it would make to our calculations if the data were to be collected by direct contact with registrants over the same time period.

Fourthly, it was not always clear-cut from the professional associations' databases in which particular countries individual psychiatrists had trained and so individuals with such incomplete data were left out of the calculations.

Fifthly, we were unable to access the data of country of origin of psychiatrists in Australia who have been recruited to work in areas of need, but who are not registered with the RANZCP. Thus our Australian data is a considerable underestimate. The process of this analysis has highlighted the difficulties in obtaining an accurate overview of brain drain. However, we believe the data provide a significant start to understanding the scale of the migration of psychiatrists from low and middle-income countries to richer ones, and its impact on population access to mental health care in the donor countries.

The ratios of psychiatrists per head of population in the west have risen dramatically (for example in the UK in the 1970s, the ratio was 1 psychiatrist per 100,000; in the 1990s it was 1 per 50,000 and now it is around 1 per 10,000). In contrast, the numbers of psychiatrists in Africa is still extremely low (0.33 per 100,000) which we have estimated from our data would have been 0.52 per 100,000 if movement to high-income countries had not occurred.

A recent World Psychiatric Association Taskforce on brain drain conducted a small survey to explore reasons for psychiatrist migration, and found that professional isolation and the search for better training opportunities were key reasons for emigrating. Another reason was the paucity of other mental health professionals resulting in the lack of a multidisciplinary approach, and poor treatment conditions for patients [32].

As an example, Nigeria's plan to integrate the delivery of mental health service into primary care has failed because of the shortage of psychiatrists. Only 19 of Nigeria's 36 states as well as the national capital, Abuja, have any psychiatrists at all. Nigeria had a well regarded home-based specialist training programme in psychiatry for over 25 years, but today this is threatened by a lack of suitably qualified psychiatrists.

Training of psychiatrists is also affected by lack of adequate numbers of trainers. Even though Nigeria has had home-based specialist training programmes in psychiatry for over 25 years, the rate of production of specialists has remained stunted, and currently, about only about 50% of Nigeria's tertiary mental health facilities have enough psychiatrists on their staff to be able to provide accredited training. If Nigerian-trained psychiatrists living overseas were to return to work in Nigeria, the country could probably double its mental health manpower every 5–6 years.

The overarching objective of global health initiatives is equity to address differences in health status that are unnecessary, avoidable and unfair; to direct more resources for health to those in greatest health need; and to influence decisions on how resources for health are shared and allocated. In relation to the inequitable global distribution of health workers, there are many political and economic reasons which encourage health workers and their families to leave poor countries to seek a better life, whether better in terms of security, education or quality of work environment. Health workers, like any other world citizen, have Labour rights, rights to education, health, non-discrimination and equality, as established by the 1947 UN Declaration of Human Rights[33]. However, ethical principles and rights are never absolute – and always needs to be tempered by competing rights [34]. Thus, the population of the source country also has a right to health, which is just as important as the right to health of the destination country, and as the individual rights of the migrant health workers[35]. Indeed, the right to health is now entrenched in international law in the 1996 International covenant on economic, social and cultural rights. This right to health is seriously undermined when health professionals decide to leave poor countries[36]. Other considerations include the poor countries loss of investment in the education and experience of those who emigrate[10]. In many developing countries, undergraduate places in medical schools are grossly short compared to the number of candidates who want them. When some of the few who eventually get those places emigrate, the loss to the country is compounded.

It has been frequently argued that, despite the damage to health systems, poor countries gain from exporting health workers by remittances sent back to the donor country. However, there is evidence from the Philippines that the intellectual and financial capital accrued to the sending country does not balance the detrimental effects of losing a much needed health worker, and that remittances are seldom used for productive public purposes, directed to the poor, or to health systems, but rather directed to the family of the health worker, which is usually already relatively better off then the bulk of the population of the donor country [37]. The Philippines have donated nurses for very many years and are now short of nurses, and wards are closed [20], [21], [38]. Meanwhile, some Filipino doctors are converting to nursing so they too can emigrate [39].

More research on migration of health workers is crucial, especially methodological developments to resolve the problems of measurement of cost benefit data, monetary values, numbers of staff, and rate at which costs should be discounted. If a senior person leaves a country, the country loses teaching skills and capacity, service development and policy dialogue as well as clinical skills. Internal migration within countries,(from rural to urban areas, and from public health systems to higher paid jobs in NGOs and private practice) also further compromises access to equitable health care [40].

Creative policy approaches are needed to ensure that individual rights of health professionals do not compromise the societal right to health. These might include career path incentives such as continuing professional development, higher training, scholarships, bonding agreements, research opportunities and flexible working, especially for women to encourage staff retention and motivation. Social incentives include provision of housing (Lesotho, Mozambique, Malawi, Tanzania), staff transport (Lesotho, Malawi, Zambia), child care (Swaziland), free food (Mozambique, Mauritius), better facilities and equipment, security for staff, HR management, access to health care and provision of medication for HIV [41].

A comprehensive approach of international agreements to mitigate harm to the supply of health workers in low and middle income countries is crucial, combined with collaborative international partnerships to strengthen general health services and specialist mental health services in low and middle income countries, and accompanied by stronger capacity for workforce monitoring and planning[8].

Migration of doctors is likely to continue until international aid incorporates a human capital and health systems strengthening agenda which respects the right to health of the general population. Task shifting to primary care nurses and clinical officers and to psychiatric nurses has already long been in place for mental health , but without adequate involvement of and systematic support from well trained specialists , the quality of training, service development, policy dialogue, assessment and treatment provided is compromised.
